# Grid Search for Lowest Root Mean Squared Error in Predicting Optimal Sensor Location in Protected Cultivation Systems

**DOI:** 10.3389/fpls.2022.920284

**Published:** 2022-07-07

**Authors:** Daniel Dooyum Uyeh, Olayinka Iyiola, Rammohan Mallipeddi, Senorpe Asem-Hiablie, Maryleen Amaizu, Yushin Ha, Tusan Park

**Affiliations:** ^1^Department of Bio-Industrial Machinery Engineering, Kyungpook National University, Daegu, South Korea; ^2^Upland-Field Machinery Research Center, Kyungpook National University, Daegu, South Korea; ^3^Smart Agriculture Innovation Center, Kyungpook National University, Daegu, South Korea; ^4^Department of Hydro Science and Engineering, Technische Universität Dresden, Dresden, Germany; ^5^Department of Artificial Intelligence, School of Electronics Engineering, Kyungpook National University, Daegu, South Korea; ^6^Institutes of Energy and the Environment, The Pennsylvania State University, University Park, PA, United States; ^7^College of Science and Engineering, University of Leicester, Leicester, United Kingdom

**Keywords:** air-vapor mixture, artificial intelligence, greenhouse, machine learning, psychrometric properties, RMSE, time-series big data

## Abstract

Irregular changes in the internal climates of protected cultivation systems can prevent attainment of optimal yield when the environmental conditions are not adequately monitored and controlled. Key to indoor environment monitoring and control and potentially reducing operational costs are the strategic placement of an optimal number of sensors using a robust method. A multi-objective approach based on supervised machine learning was used to determine the optimal number of sensors and installation positions in a protected cultivation system. Specifically, a gradient boosting algorithm, a form of a tree-based model, was fitted to measured (temperature and humidity) and derived conditions (dew point temperature, humidity ratio, enthalpy, and specific volume). Feature variables were forecasted in a time-series manner. Training and validation data were categorized without randomizing the observations to ensure the features remained time-dependent. Evaluations of the variations in the number and location of sensors by day, week, and month were done to observe the impact of environmental fluctuations on the optimal number and location of placement of sensors. Results showed that less than 32% of the 56 sensors considered in this study were needed to optimally monitor the protected cultivation system’s internal environment with the highest occurring in May. In May, an average change of −0.041% in consecutive RMSE values ranged from the 1st sensor location (0.027°C) to the 17th sensor location (0.013°C). The derived properties better described the ambient condition of the indoor air than the directly measured, leading to a better performing machine learning model. A machine learning model was developed and proposed to determine the optimal sensors number and positions in a protected cultivation system.

## Introduction

The changing climate and depletion of natural resources such as fossil-based energy, land, and water necessitate improving resource use efficiency. Protected cultivation systems such as greenhouses could be essential in efficiently providing nutritious fresh foods for a growing world population (Stanghellini, 2013). Higher water use efficiency per unit area of crop production has been recorded in protected cultivation systems compared to open-field cultivation (Li et al., 2010). This could be a potential solution to land scarcity. Where disasters such as pandemics make farms momentarily less accessible, remotely controlled and autonomous cultivation strategies would be beneficial.

However, the benefits in these systems come at higher energy demands, especially when poor decisions are made based on incorrect monitoring of the micro-climate. Overheating and consequently poor plant growth and ensuing economic losses could be one such result (Park and Park, 2011). Protected cultivation systems could, however, be capital intensive. Improved efficiency will reduce the system’s energy consumption and reduce production costs (DeFacio et al., 2002; Vox et al., 2010).

In protected cultivation systems, irregular changes or high fluctuations in indoor climatic conditions can be deleterious to productivity. Temperature and relative humidity management to meet specific plant requirements is critical for survival, optimum growth, and enhanced productivity (DeFacio et al., 2002; Vox et al., 2010). The optimal placement of the minimum number of sensors for measuring the micro-climate of protected cultivation systems is critical for their efficient use and sustainability. The protected cultivation system has a high level of variability caused by plant respiration and heating systems.

Ventilation causes air movement and consequently the uniformity of the environment. In Guzmán et al. (2019), the wind direction was reported to have a significant effect on ventilation rate, airflow, and crop temperature distributions. Also, in Li et al. (2010), it was observed that temperature did not rise linearly between inlet and fans and was higher at or above the top of the crop canopy than within it in a full-size house but not in a glasshouse compartment. A method for determining the optimal number and locations of the sensors would be necessary to accurately measure the environment of a protected cultivation system.

Recent high-tech protected cultivation systems are equipped with advanced sensors for monitoring parameters such as temperature, relative humidity, CO_2_, and light. This is done to improve monitoring and control of micro-climate parameters and sometimes facilitate remote-controlled and autonomous cultivation. Decisions may be made based on various actuators used to regulate heating, lighting, cooling, dosing of CO_2_ and fertilizers, dehumidification, irrigation, screening, fogging, as examples (Nelson, 1991; Uyeh et al., 2019, 2021; Bhujel et al., 2020; Gadekallu et al., 2021). These actuators operate based on sensors providing feedback on measured data for the control loop set points configured in a computing device (Stanghellini, 2013; Graamans et al., 2018).

In autonomous growing systems (Stanghellini, 2013; Graamans et al., 2018; Hemming et al., 2020), deployment of the more costly, high-precision sensors have added benefits such as durability and reduced capital costs in the long-term. Decisions based on imprecise measurements could result in poor plant growth (due to under-or over-heating) or irreversible damage and associated economic losses. An additional benefit of using more precise sensors is energy savings.

Growers constantly face decision-making and optimization problems in agriculture. Multiclass models have been used to develop multivariate statistical methods in agriculture (Guzmán et al., 2019) and Principal Component Analysis - whale optimization-based neural networks to classify diseases in plants (Li et al., 2010). Others include algorithms and systems for improved decision-making and optimizations (Nelson, 1991; DeFacio et al., 2002; Vox et al., 2010; Park and Park, 2011; Uyeh et al., 2019; Gadekallu et al., 2021). Machine learning provides opportunities to solve complex tasks such as optimal sensor placement because of its capabilities to efficiently compute vast and complex datasets with a high success ratio and fewer errors (Syed and Hachem, 2019a,b).

To solve the optimal sensors placement problem, this study, (a) designed and fabricated temperature and humidity sensors to monitor every section of a protected cultivation system and accurately collect data per minute were, (b) derived psychometric properties to understand better, the actual condition and behavior of the air-vapor mixture in a protected cultivation system, and (c) proposed a machine-learning solution based on the derived psychometric properties.

A machine learning algorithm, the Gradient Boosting Algorithm, was implemented as a multi-objective approach to determine the optimal number of sensors and locate their best position. The objective function of this algorithm was to minimize the root mean squared error (RMSE) and the number of sensors using two multiple hyper-parameter tuning algorithms (Random Search and Grid Search).

### Related Works

Growers constantly face decision-making and optimization problems. Multiclass models have been used to develop multivariate statistical methods in agriculture (Gadekallu et al., 2021) and principal whale optimization-based neural networks to classify diseases in plants (Gadekallu et al., 2021). Others include algorithms and systems for improved decision-making and optimizations (Park et al., 2019; Syed and Hachem, 2019a,b; Uyeh et al., 2019, 2021; Bhujel et al., 2020). Using an inadequate number of sensors may lead to under-performance, while a likely result of being superfluous is large sizes of redundant data and its associated management problems. The sensor placement problem has been recognized and studied in other fields. These include fire detection in a target region (Li et al., 2013), air and water quality monitoring (Du et al., 2014; Fontanini et al., 2016), and monitoring physical activity in humans with a three-dimensional accelerator (Boerema et al., 2014). Others include structural health monitoring based on modal data (Chang and Pakzad, 2014; Tong et al., 2014) and mid and low frequency range methods (Rao et al., 2014). Attempts have been made to determine the optimal sensor selection and location in internal environments, focusing on structures stability (Worden and Burrows, 2001; Löhner and Camelli, 2005; Wang et al., 2009; Chang et al., 2012; Hu and Patel, 2014; Huang et al., 2014; Arnesano et al., 2016; Seabrook, 2016). Worden and Burrows (2001) studied the optimal temperature sensor location using an error-based approach for monitoring a stadium’s heating, venting, and air-conditioning systems.

As the environment in protected cultivation systems is dynamic, optimal sensor placement may involve the following scenarios: (a) multiple sensor types required in one system with two or more sometimes embedded as one (Faris and Mahmood, 2014); (b) movements of the rising and setting sun which affects the internal data (Cossu et al., 2014; Wang et al., 2014); (c) multiple layers of plant beds with varying atmospheric conditions at each level (Pamungkas et al., 2014); and (d) the influence of other internal structures of the system.

Techniques for selecting and installing sensors for monitoring and controlling climatic conditions in protected cultivation systems such as plant factories, greenhouses, etc., have been mostly heuristic. Feng et al. (2013), simulated greenhouse internal air temperature and wind-velocity distributions and suggested that the optimal sensor location is where the air and speed do not change rapidly. Several approaches, such as z-index, the outliers, and statistical measures, including central tendency and dispersion measures, have been employed (Lee et al., 2019). This study’s limitation was the low volume of air temperature data and the non-inclusion of other influencing environmental variables such as humidity and light. In more complex and larger-sized systems, statistically based techniques incapable of handling big data would be ineffective.

Some studies attempted to use machine learning to determine the number of optimal sensors and identify their locations (Aydin et al., 2019), however, derived conditions (dew point temperature, humidity ratio, enthalpy, and specific volume), or some other environmental variables were not taken into consideration to provide a better representation of the protected cultivation system state. According to Ponce et al. (2014), most analytic models focusing on controlling the internal environment of protected cultivation systems have been based on a state-space relationship. This state-space form includes variables such as indoor temperature, humidity, energy input, outdoor temperature, wind speed, time, etc. Further, they (Ponce et al., 2014) recorded temperature and humidity are influential variables used to simplify the greenhouse state. Psychometric properties such as dew point temperature, humidity ratio, enthalpy, and specific volume would be beneficial to better represent the greenhouse’s dynamic behavior, especially since air is mixed with vapor (Czubinski et al., 2013).

## Methodology

***a. Overview***

Temperature and relative humidity data were collected remotely from a protected cultivation system located on the research farm of Kyungpook National University, South Korea. Data was collected over seven months (February, March, April, May, June, July, and October). The time-series observation for the two conditional parameters recorded per minute were representative data.

**FIGURE 1:**
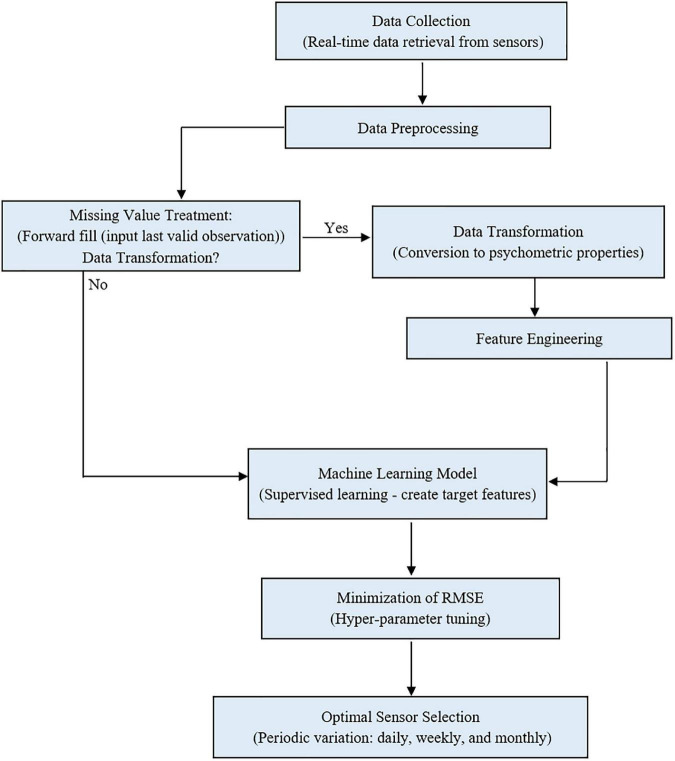
Workflow for optimal sensor selection using (state method) in a protected cultivation system.

The temperature and humidity data were preprocessed, and four psychometric variables (dew point temperature, humidity ratio, enthalpy, and specific volume) were derived and used to model the protected cultivation system’s indoor environment. The algorithm was trained on 70 % of the data to ensure generalization and no overfitting. The metric of evaluation, RMSE, was minimized by tuning the algorithm’s hyper-parameters (parameters whose values are used to alter the machine learning algorithm’s learning rate) iteratively. Based on each month, sensor ranking was carried out. Furthermore, the number of optimal sensors required daily, weekly, and monthly was determined in a supervised manner. Figure 1 shows the workflow for optimal sensor selection. The data is collected using the fabricated temperature and humidity sensors and stored in a cloud system. The collected data was preprocessed using forward fill and transformed into psychrometric variables. The preprocessed temperature, humidity, and transformed psychrometric data were used to develop the supervised machine learning model. Optimal sensor selection was done by minimizing RMSE using hyper-parameter tuning.

***b. Experiment setup and protected cultivation system location***

**FIGURE 2:**
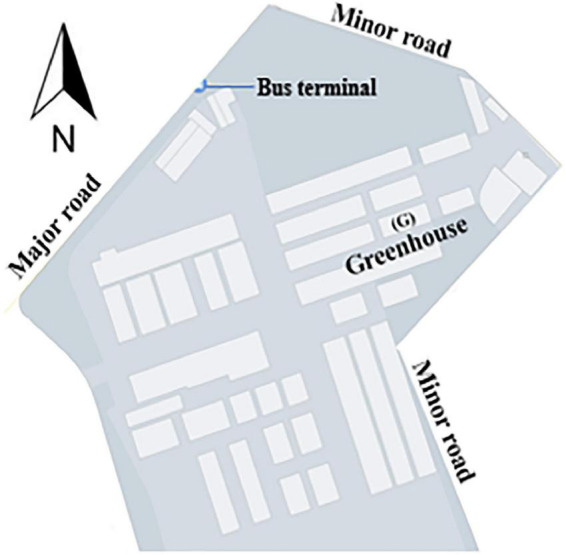
Location of the experimental greenhouse (G) used for data collection for optimal sensor placement study.

A Quonset-shaped protected cultivation system (greenhouse) located on the research farm of Kyungpook National University, Daegu, South Korea (35°53′43.0 N and 128°36′49.1 E) was selected for this study. The greenhouse is used to cultivate strawberries and is close to two inner roads and a major road with heavy vehicular traffic (Figure 2).

**FIGURE 3:**
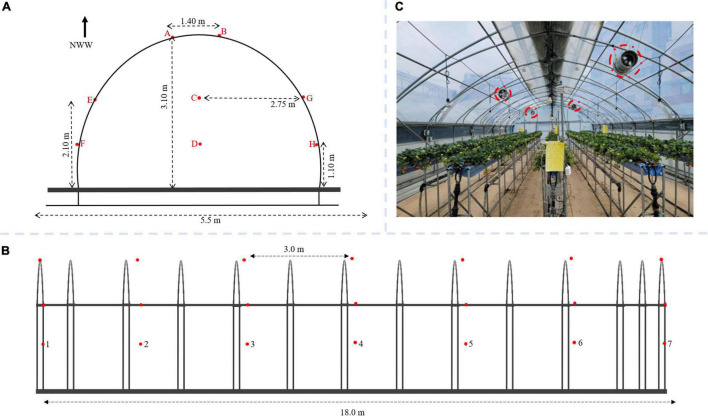
The experimental greenhouse with temperature and relative humidity sensors installed; **(A)** front view; **(B)** side view for optimal sensor placement study; and **(C)** with growing strawberry plants and fan for mechanical ventilation circled in broken red lines.

Fifty-six 2-in-1 temperature and humidity sensors were installed on eight rows and seven columns, each at 3 m horizontal and 1 m vertical distance apart for uniformity (Figure 3). The sensors were specifically manufactured to have a similar range (and error) of −20°C to 80°C (± 0.3°C) and 0% to 100% (± 2%) for temperature and relative humidity, respectively. The sensors were installed in different columns represented with A – H (Figure 3A) and seven fixed rows in Figure 3C. To prevent solar radiation from interfering with readings and causing errors, the sensors were enclosed in a plastic covering. Constantly running ventilation fans were installed in the greenhouse (Figure 3B).

***c. Environmental sensing of protected cultivation system and data collection***

**FIGURE 4:**
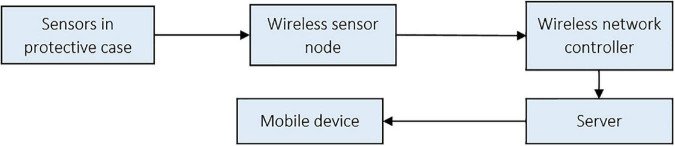
Wireless system architecture for remote sensing of the protected cultivation system.

A network controller (U-NWC-W-7S, UBN, Daegu, South Korea) was installed to minimize the temperature and relative humidity data collection error from the 56 sensors. The controller has a distributed processing system, a radio frequency of 447.9 MHz, enabling mobile software development for real-time data retrieval from the sensors. The sensor-controller system’s architecture is shown in Figure 4.

The sensors were tightly installed to prevent movement and connected via cables to the sensor nodes, which transferred data *via* gateways to a server and then to a mobile telephone device. This wireless system enabled consistent remote monitoring. Preventive maintenance of the systems was regularly carried out to avert errors from factors such as sensor clogging.

***d. Variability analysis of greenhouse environmental data***

The variability of the conditions within the greenhouse was measured by calculating the Coefficient of Variation (*CV*) as the ratio of the standard (Equation 1) deviation to the mean temperature/humidity in each period (when expressed as a percentage) as used by Ayalew et al. (2012) and Kassie (2014).


(1) $$\mathrm{Coefficient}\mathrm{of}\mathrm{Variation}\left(\mathrm{CV}\right)=\frac{\mathrm{Standard}\mathrm{deviation},\sigma }{\mathrm{Mean},\mu }$$

***e. Dynamic time warping to determine the effect of the plants on microclimate distribution***

*i. Data Description*

Using the hourly reading of the temperature and relative humidity data collected in March with plants and June when the greenhouse was without plants, the data dimensions for March and June were 744, 113, and 720, 113, respectively.

*ii. Implementation of dynamic time warping algorithm*

The dynamic time warping (DTW) algorithm, following Furlanello et al. (2006) and given below, was implemented to ascertain the effect of the plants on the microclimate distribution of the greenhouse.

*Input:*

series: u = {*u*_1_, *u*_2_,…, *u*_*T_u_*_}

series: v = {*v*_1_, *v*_2_,…, *v*_*T_v_*_}

*Base conditions :*

g (0,0) = 0

g (1,1) = d (*u*_1_, *v*_1_)⋅*w*_*D*_

g (i,0) = ∞ for 1 ≤ i ≤ *T_u_*

g (0, j) = ∞ for 1 ≤ j ≤ *T_v_*

*Recursive relation:*


$$\text{g}\left(\text{i},\text{j}\right)=\text{min }\{\begin{array}{c} g(i,j-1)+d(u_{i},v_{j})\cdot w_{v}\\ g(i-1,j-1)+d(u_{i},v_{j})\cdot w_{D}\\ g(i-1,j)+d(u_{i},v_{j})\cdot w_{H} \end{array}$$

for 1 ≤ i ≤ *T_u_* and 1 ≤ *j* ≤ *T_v_*

Alignment deduction by tracing back from g (*T_u_, T_v_*) to g (0,0).

Where *T_u_* and *T_v_* are the time points for series u and v, respectively; d is the local distance minimized by the DTW algorithm to find the minimum cost path or best alignment; g is the matrix of the dynamic table construction of (*T_u_* + 1) × (*T_v_* + 1); *w_H_*, *w_D_*, *w_V_* are the weight configuration for horizontal (H), diagonal (D) and vertical (V) time distortions.

***f. Protected Cultivation Environmental Data Preprocessing***

Preprocessing the data involved standardizing features (sensor locations) and treating missing values. To standardize the features within a range of 0 to 1, feature scaling was done. Train-validation split was carried out in a time series to avoid a randomized or highly stochastic output. The tree-based algorithm (Gradient Boosting) was fitted on the training data and validated on the remaining (or unseen) portion to prevent overfitting.

**FIGURE 5:**
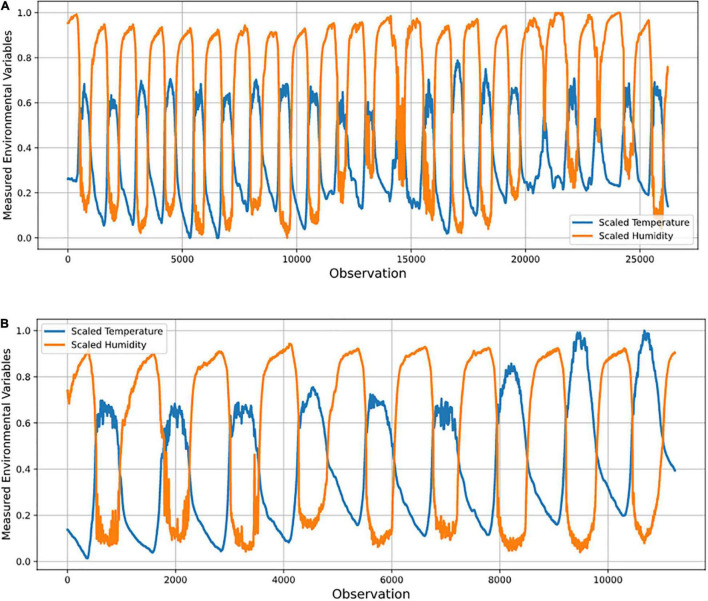
Graphs of **(A)** training data; and **(B)** test data used in data preprocessing for sensor A1 in optimal sensor placement study.

Missing data was less than 1%, and these were treated with forward (or backward) filling given the appropriateness of this approach for the observations recorded within a minute. Figure 5 shows the result of the data preprocessing at sensor A1.

From the two condition parameters – temperature and humidity – psychometric properties (dew point temperature, humid ratio, enthalpy, and specific volume) describing the air vapor mixture (Czubinski et al., 2013) in the greenhouse were derived. This helped to determine more features of importance as condition parameters for the greenhouse environment.

***g. Derivation of Psychometric Variables***

Equations 2–5 were used to convert the raw temperature and relative humidity data into dew point temperature, humidity ratio, enthalpy, and specific volume (Handbook, 2001):


(2) Dewpointtemperature(C°),Td=T-(100-RH)5

(3) $$\mathrm{Humidity}\mathrm{ratio},w=\frac{0.62198P_{w}}{P-P_{w}}$$

(4) Enthalpy(kJ/kg),h=1.006T+w(2501+1.805(T))

(5) Specificvolume(m/3kg),v=RdaTP-Pw

Where *T* was internal temperature; *RH*, relative humidity; *P*_*w*_, partial pressure of water vapor; *P*, total pressure; and *R*_*da*_, gas constant for dry air = 287.055 J/(kg K).

### Optimal Sensors Placement Problem Formulation

***Objective 1: Minimizing the RMSE (Sensor Location Ranking)***

A single sensor location that gives the maximum gain to the objective function (Equation 6) was selected from all the environment’s 56 possible positions. Furthermore, having fixed the previous selection of the best sensor location, the following location was determined from the remaining (56 – 1 = 55) locations that gave the best improvement in the objective – lowest RMSE. This technique was applied iteratively until the last sensor location was determined. That is, *RMSE*_*min*,1_, *RMSE*_*min*,2_, *RMSE*_*min*,3_,…, *RMSE*_*min*,56_, where 1, 2,…, 56 are placeholders for the sensor nodes, A1, A2,…, H7 (not necessarily in this order but ranked by the minimum RMSE at each node).


(6) RMSEmin_N=∑i=1n(xi(i,t)-x^i(i,t))2n

Where N was sensor location number, *x*_*i*_(*i, t*) is the actual observation of the climatic variables at location *i* and time *t*, x^i(i,t) was the estimated value, and *n* was the total number of nodes or sensor locations.

N submatrices of the matrix, **A** of *m* × *n* representing the data, such that, **A** ∈ *R*^*m* × *n*^ were derived to represent the observations at each sensor node given that *S*_*m*,*p*→*q*_ ∈ *R*^*m* × *p*→*q*^ ∀ m, n, p, q ∈ N {p, q < n}. *p*→*q* took an element from the start of the column of a particular sensor location to the end of the column (a node was defined at column index p and q-1 with temperature and humidity index, respectively, for a two-in-one sensor). Sub matrix, *S* of elements *a*_*ij*_ where i = 1, 2, …, m; j = 1, 2, …, q-1 is ordered in a rectangular frame as shown in Equation 7.


(7) $$S=\left[a_{1p}a_{1q-1}a_{2p}a_{2q-1}\vdots \vdots a_{mp}a_{mq-1}\right],a_{ij}\in R$$

A supervised learning approach was employed for this study. As such, a response or target variable (climatic variables to be predicted by the input features), *y*_*t+k*_ was derived by making a *k*-step forecast of a column (feature) of the submatrix, *S* for *k* ∈ N. All observations were made per minute and the response variable at time t was one step ahead of the observation; thus, *k* = 1. A machine learning model simply represented in Equation 8 where d = 1, 2, … D were index features, was fitted on the new data matrix, *C* ∈ R^*m* × 3^ ⊇ *S* and evaluated by the performance metric, RMSE. This was carried out for all sensor nodes, and the RMSEs were used to rank the order of importance of the sensor nodes – in the order of increasing RMSE values. This implied a larger improvement to the objective function is used to rank the sensors.


(8) $$f:R^{D}\to R$$

***Objective 2: Minimizing the Optimal Number of Sensors***

The second objective of this study was to determine the minimum optimal number of sensors and the sensor location ranking. Having determined the sensor location that gave the most considerable improvement to the objective function, the target variable, *y*_*t+k*_ was taken to be a one-step forecast of one of the environmental variables (temperature) readings at this location. Following Li (2016), a gradient boosting model at each point *m* of *M* stages, *G_m_* such that 1 ≤ *m* ≤ *M* was fitted on the preprocessed data, with the subsequent addition of some estimators, *h*_*m*_(*x*)(regression trees) to improve the model by compensating for the inadequacy of the existing model *G*_*m*_(*x*) (Equation 9).


(9) $$G_{m1}\left(x\right)=G_{m}\left(x\right)+h_{m}\left(x\right)$$

*G*_*m*1_(*x*) is the new model, *G*_*m*_(*x*) is the existing model and *h*_*m*_(*x*) is the regression tree.

As a supervised learning problem for the training data, {(*x*_1_, *y*_1_), …, (*x*_*n*_, *y*_*n*_)}, an approximation function, G^(x) extended a function *G*(*x*) to minimize the objective function given as *R*(*y*_*t*+*k*_, *G*(*x*)) by starting with a model containing function *G*_0_(*x*) and expanding the model as given in Equations 10 and 11.


(10) $$G_{0}(x)=\;arg\;\underset{ţ}{min}\sum_{i=1}^{n}R(y_{i},\;\text{μ})$$

(11) $$G_{m}\left(x\right)=G_{m-1}\left(x\right)+\left[\sum_{i=1}^{n}R\left(y_{i},G_{m-1}\left(x_{i}\right)+h_{m}\left(x_{i}\right)\right)\right]$$

where *i* ∈ *N*, *h*_*m*_ ∈ *H* is a base learner function.

The model was updated by applying the steepest gradient descent to the minimization problem in Equations 12 and 13.


(12) $$G_{m}\left(x\right)=G_{m-1}\left(x\right)-\mu_{m}\sum_{i=1}^{n}\nabla_{G_{m-1}}R\left(y_{i},G_{m-1}\left(x_{i}\right)\right)$$

(13) $$\mu_{m}=\sum_{i=1}^{n}R\left(y_{i},G_{m-1}\left(x_{i}\right)-\mu \nabla_{G_{m-1}}R\left(y_{i},G_{m-1}\left(x_{i}\right)\right)\right)$$

where the derivatives are taken concerning the functions *Gi* for *i* ∈ {1, , *m*} and _*m*_ was the step length.

**FIGURE 6:**
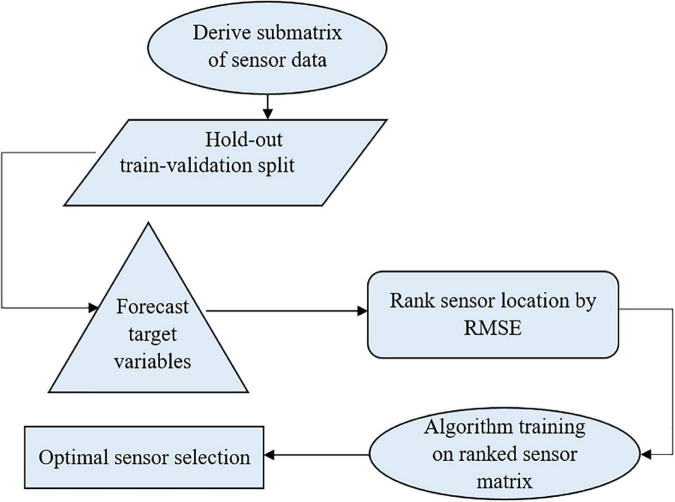
Flow chart showing the summary of the model building process for optimal sensor selection.

An *N* number of sensor locations was considered, where *RMSE*_*min*,1_ < *RMSE*_*min*,2_ < *RMSE*_*min*,3_ < *RMSE*_*min*,4_ < … < *RMSE*_*min*,*N*_ for each node from 1, 2, …, *N*. A one-step-ahead time forecast at node 1 was taken as the response or target variable to be used as a predictor for other nodes to determine the performance of placement, while, for the first aspect, the input features were environmental variables (temperature and humidity), and the second aspect, the four psychometric properties (dew point temperature, humid ratio, enthalpy, and specific volume), plus crucially engineered features of the date/time object variable. The overall RMSE continued to decrease, indicating improvements in the sensor stacking performance until a point was reached where there was no further minimization of the objective function. At this point, the number of sensors was considered as being optimal. The pseudo-code below illustrates the algorithm for optimal sensor selection, and Figure 6 shows the summary of this process. The algorithm used flow conditional statements that iterated the whole process of ranking. The RMSE was the objective function. It was the metric for evaluating the variability due to the disturbances in the greenhouse’s climate. The ranking was done by using a time-series forecast methodology. The RMSE compared the predicted values with the actual values.

The RMSE was minimized by tuning the hyper-parameters of the algorithm to obtain the best result. This also ensured that the ranking was not subjected to fluctuations and the optimal selection was accurate no matter how many times the pipeline was automated/re-run.

**Table:** Optimal sensor selection pseudo-code.

**Input:** Temperature-humidity dataset, A ∈ ℝ^*m* × *n*^, *of*(*m* × *n*) dimension Output: Set of optimal sensors **Ranking of…** **Ranking of sensors** 1 Create data-frame for each sensor location 2 Split into *X*_*train*_ and *X*_*val*_ in a time-series manner 3 Derive target variable by forecasting a feature’s observations 4 Split into *y*_*train*_ and *y*_*val*_ 5 Fit the model on the data 6 Append sensor locations to a list by RMSE ranking (Update the list of sensors iteratively with the corresponding RMSE values in an ascending order to show ranking) Optimal number of sensors 7 Initialize optimal sensors (p) to 0 8 Assign v to 56 (total number of sensors) 9 for i = 1 to v do 10 Fit model on training set and score 11 if hyper-parameter is not optimal do 12 try other combinations of hyper-parameters 13 else do 14 append RMSE values [Equation 6] 15 increment p by 1 16 return p


## Experimental Results and Discussion

In (Lee et al., 2019), a statistical approach was adopted for optimal sensor selection. Our study advanced the optimal sensor selection by developing a machine learning model using generated time-series big data and transformed psychrometric variables. In the results obtained in Lee et al. (2019), sensor locations with the highest entropy were selected as optimal because of high disturbance from the wind. We implemented an algorithm on time-series big data and transformed psychrometric variables that choose sensors that can best monitor the state of the greenhouse optimally using hyper-parameter tuning.

### Variability Analysis: Coefficient of Variation of Greenhouse Temperature and Humidity Data

The coefficient of variation (*CV*) of the climate data was calculated for all the studied months. The *CV* was used to determine the extent of variability of the greenhouse by computing the ratio of the standard deviation to the mean of the temperature or relative humidity values.

During the summer period (June and July) in Table 1, it was observed that the temperature variation was the least, indicating the data points have the minimum difference from the mean compared to other periods. July showed the least CV for the relative humidity data but differed slightly from June and showed a slightly higher value than February. Similarly, the greenhouse had the least variability in the summer months for relative humidity. Considering plants were not grown during this period in the greenhouse could be a reason for the low variation in greenhouse climate properties. During aerobic respiration, plants use oxygen and emit carbon dioxide (Kader and Saltveit, 2002), which affects the properties of the greenhouse. Generally, this was observed in other months in which plants were grown.

**TABLE 1:** Coefficient of Variation for temperature-relative humidity data for estimating the variability of the greenhouse.

Month	Temperature CV (%)	Relative humidity CV (%)
June	22.1	36.70
October	25.26	38.96
February	40.43	32.53
July	14.08	19.30
March	42.30	42.09
May	24.65	38.31

February and March (the end of winter and the beginning of spring) are the two months with the highest temperature *CV* (40.43% and 42.30%, respectively), similarly with a very high humidity *CV* (32.53% and 42.09%, respectively). This was probably caused by the changing season, with a sharp change in weather conditions. February and March recorded a low of −9°C and −2°C, respectively. Both months had a high of 24°C. An increment of 9.15% and 30.94% in the temperature and humidity standard deviation, respectively, were observed in March.

### The Effect of the Plants on the Microclimate Distribution

**FIGURE 7:**
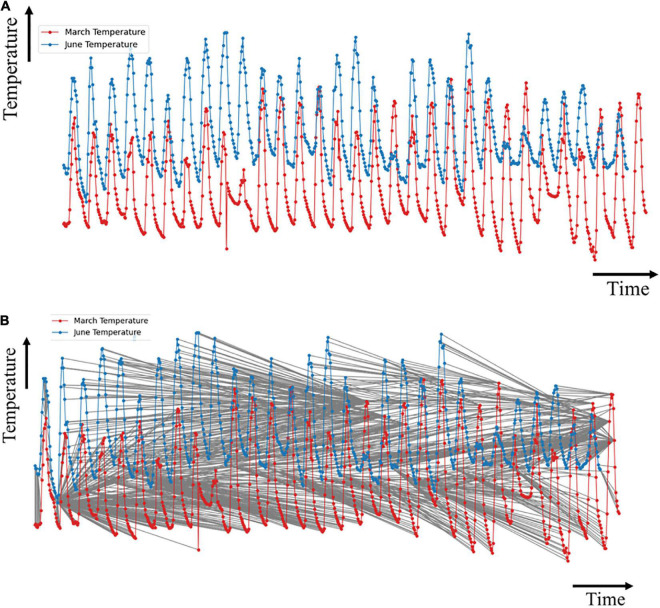
Plots of temperature data for **(A)** March and June; and **(B)** optimal match of the time series.

The dynamic time warping algorithm was implemented to measure the similarity between March and June sensor readings. Figure 7A shows the plot of the March and June sensor reading per hour for the temperature data, and Figure 7B shows the alignment match plot of the series. The optimal match between the two series, as shown in Figure 7B, cannot be understood visually since the dataset is quite large.

The first index from the *i* sequence matches with at least 250 indices of the *j* sequence. This implies that the first hour of June matches with the first 11 days of March with a minimal cost path as indicated by the vertical line. This implied that there were no statistically significant changes in the climatic condition of the greenhouse. However, before the end of the first day in June, a slow change in the graph indicated a shift of alignment between the two months. A significant match occurred at the 170th index of the *j* sequence (7th day of June) with the 352*^nd^* index of the *i* sequence (toward the evening of the 14th day of March).

This similarity was stable for about 48 h (2 days). The overall climatic condition of the greenhouse, by the temperature, in the last seven days in June matched closely with the state of the greenhouse within the previous two days of March, with minor variations.

Almost all points of the *i* and *j* indices had unique matches for the relative humidity data. A lesser number of matches of the indices was observed. This high linearity implied that the absence of crops in June did not have much effect on the relative humidity of the greenhouse compared to the temperature. This also justified a lesser percentage decrease from March to June in the relative humidity *CV* than the *CV* of temperature as shown in Table 1.

### Temperature-Relative Humidity Data

The optimal hyper-parameters were *n*_*estimators* = 1000 and *learning*_*rate* = 0.01, while *max*_*depth* ranged from 2 to 7, as selected by the iterative algorithm. These hyper-parameters were used to tune the algorithms to learn the data with the maximum performance. Seven months (February, March, April, May, June, July, and October) were selected as representative months to cover the four seasons (winter, spring, summer, and autumn) and used in the simulations.

In Table 2, index numbers 3, 5, 12, 4, 3, 8, and 1 with the least RMSE values of 0.0102077, 0.0194982, 0.0223317, 0.0171915, 0.0103634, 0.0046052, and 0.0355036 were recorded as optimal sensors numbers and locations for February, March, April, May, June, July, and October, respectively, for temperature data. At some months, the RMSE values start increasing, indicating that the addition of more sensors would instead reduce the quality of the data. These presented the sensors that measured the air-moisture condition in the greenhouse most accurately in the different months. The sensors acted as features or variables used for training the machine learning model. Through ranking, the number of sensors required was determined with the RMSE indicating the model’s performance in predicting the best sensor location. The more relevant the feature(s), the lower the RMSE. The row (bolded) beyond which the RMSE no longer decreased was taken as the optimal. Table 2 shows index number that a high variation in the optimal number of sensors occurred at different months with a total number of 4, 6, 13, 5, 3, 9, and 3 sensors were optimal for measuring the greenhouse’s internal environment in February, March, April, May, June, July, and October, respectively.

**TABLE 2:** Performance of a sensor network in identifying the optimal number of sensors and placement for measuring greenhouse conditions across different months using temperature data.

Index	Sensor location (s)	RMSE (°*C*)
**February (F7)**		
0	C2	0.0448124
1	C2, H7	0.0133850
2	C2, H7, B7	0.0114320
3	C2, H7, B7, A1	**0.0102077**
4	C2, H7, B7, A1, D6	0.0108046
5	C2, H7, B7, A1, D6, F1	0.0110145
**March (G7)**		
0	H7	0.0380515
1	H7, D5	0.0278228
2	H7, D5, F7	0.0239464
3	H7, D5, F7, A7	0.0215741
4	H7, D5, F7, A7, B7	0.0196743
5	H7, D5, F7, A7, B7, B4	**0.0194982**
6	H7, D5, F7, A7, B7, B4, C7	0.0199467
7	H7, D5, F7, A7, B7, B4, C7, E7	0.0200142
**April (F7)**		
0	D6	0.0397207
1	D6, D7	0.0349923
2	D6, D7, F6	0.0288990
3	D6, D7, F6, F4	0.0286996
4	D6, D7, F6, F4, H7	0.0285790
5	D6, D7, F6, F4, H7, G7	0.0270916
6	D6, D7, F6, F4, H7, G7, F5	0.0268920
7	D6, D7, F6, F4, H7, G7, F5, F3	0.0266292
8	D6, D7, F6, F4, H7, G7, F5, F3, E6	0.0260851
9	D6, D7, F6, F4, H7, G7, F5, F3, E6, E7	0.0226352
10	D6, D7, F6, F4, H7, G7, F5, F3, E6, E7, H6	0.0224960
11	D6, D7, F6, F4, H7, G7, F5, F3, E6, E7, H6, D2	0.0224442
12	D6, D7, F6, F4, H7, G7, F5, F3, E6, E7, H6, D2, D5	**0.0223317**
13	D6, D7, F6, F4, H7, G7, F5, F3, E6, E7, H6, D2, D5, C6	0.0226247
14	D6, D7, F6, F4, H7, G7, F5, F3, E6, E7, H6, D2, D5, C6, D3	0.0226412
**May (D4)**		
0	A2	0.0291603
1	A2, B2	0.0288598
2	A2, B2, B3	0.0266414
3	A2, B2, B3, F1	0.0235686
4	A2, B2, B3, F1, D3	**0.0171915**
5	A2, B2, B3, F1, D3, H1	0.0172351
6	H7, D5, F7, A7, B7, B4, C7	0.0173482
**June (D5)**		
0	D4	0.0115102
1	D4, D3	0.0105300
2	D4, D3, C6	**0.0103634**
3	D4, D3, C6, C2	0.0104309
4	D4, D3, C6, C2, C4	0.0106421
**July (D4)**		
0	D5	0.0069262
1	D5, F1	0.0062005
2	D5, F1, D6	0.0061786
3	D5, F1, D6, D3	0.0051103
4	D5, F1, D6, D3, C4	0.0050485
5	D5, F1, D6, D3, C4, D2	0.0050180
6	D5, F1, D6, D3, C4, D2, C6	0.0048374
7	D5, F1, D6, D3, C4, D2, C6, F7	0.0047972
8	D5, F1, D6, D3, C4, D2, C6, F7, E1	**0.0046052**
9	D5, F1, D6, D3, C4, D2, C6, F7, E1, H7	0.0046170
10	D5, F1, D6, D3, C4, D2, C6, F7, E1, H7, F4	0.0046221
**October (B7)**		
0	B1	0.0397534
1	B1, D3	**0.0355036**
2	B1, D3, B2	0.0373681
3	B1, D3, B2, F1	0.0381142

***NB:** Sensor location in parenthesis implies the location with the least RMSE value - most important (predictor). The bolded values represent optimal sensor locations.*

**FIGURE 8:**
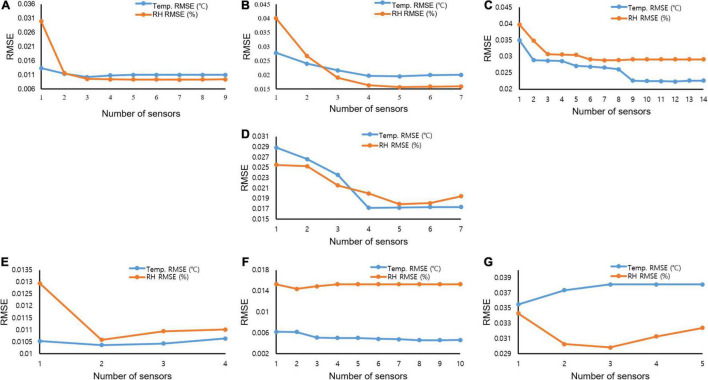
Root mean squared error curves showing the reduction in error at different numbers of sensors using temperature and relative humidity data for **(A)** February; **(B)** March; **(C)** April; **(D)** May; **(E)** June; **(F)** July; and **(G)** October.

Furthermore, investigation of the Pareto front, a set of nondominated solutions chosen as optimal when no objective can be improved without sacrificing at least one other objective, helped enhance decision-making. The two conflicting objectives showed a reduction in RMSE values at all the investigated months with increasing selected sensors. However, to reduce the RMSE and number of conflicting sensors, the Pareto front displayed the knee points where a less significant RMSE occurred. In February, (Figure 8A), a drastic reduction (about 66%) in the RMSE value between one and two sensors with a slighter decrease between two and five sensors using the temperature data. This indicated that, for February, two sensors would give good readings to understand the condition of the air-vapor mixture in the greenhouse at a less computational cost than three (about 0.014%), four, and five sensors (about 13%). This trend was seen for the other simulated months, with March (Figure 8B) having three knee points at two and five sensors with about 26% and 46%, respectively. In April (Figure 8C), two distinct knee points were recorded at three sensors (about 28%) and ten sensors (about 42%). A similar trend was seen in May (Figure 8D), June (Figure 8E), and July (Figure 8F). However, in October, a drastic reduction was seen at two sensors (about 30%), followed by a sharp rise indicating that more sensors introduced more errors instead (Figure 8G).

Index numbers 6, 4, 6, 4, 1, 1, and 2 with the least RMSE values of 0.0092886, 0.0156854, 0.0288381, 0.0179418, 0.0105844, 0.0143873, and 0.0298292 were recorded as optimal sensors numbers and locations for February, March, April, May, June, July, and October, respectively, as the sensors that measured the air-moisture condition in the greenhouse most accurately in the other months using the relative humidity data. The results for the sensors to measure the air-vapor mixture in the greenhouse differed from the temperature and humidity data. This led us to investigate the stability of the transformed data would best describe the air-vapor mixture condition in the greenhouse. In the case of relative humidity, a similar trend of high variation in the optimal number of sensors occurred at different months, with a total number of 7, 5, 7, 5, 2, 2, and 3 sensors being found optimal for measuring the internal greenhouse environment in February, March, April, May, June, July, and October, respectively (Table 3).

**TABLE 3:** Performance of sensor network in identifying the optimal number of sensors and placement for measuring greenhouse conditions across different months using relative humidity data.

Index	Sensor location(s)	RMSE (%)
**February (F7)**		
0	C2	0.0299539
1	C2, H7	0.0115791
2	C2, H7, B7	0.0095410
3	C2, H7, B7, A1	0.0094043
4	C2, H7, B7, A1, D6	0.0093536
5	C2, H7, B7, A1, D6, F1	0.0093313
6	C2, H7, B7, A1, D6, F1, C3	**0.0092886**
7	C2, H7, B7, A1, D6, F1, C3, D3	0.0093494
8	C2, H7, B7, A1, D6, F1, C3, D3, F2	0.0094121
**March (G7)**		
0	H7	0.0399899
1	H7, D5	0.0266852
2	H7, D5, F7	0.0190371
3	H7, D5, F7, A7	0.0163404
4	H7, D5, F7, A7, B7	**0.0156854**
5	H7, D5, F7, A7, B7, B4	0.0158632
6	H7, D5, F7, A7, B7, B4, C7	0.0159132
**April (F7)**		
0	D6	0.0397690
1	D6, D7	0.0347859
2	D6, D7, F6	0.0307091
3	D6, D7, F6, F4	0.0305697
4	D6, D7, F6, F4, H7	0.0304953
5	D6, D7, F6, F4, H7, G7	0.0290896
6	D6, D7, F6, F4, H7, G7, F5	**0.0288381**
7	D6, D7, F6, F4, H7, G7, F5, F3	0.0289097
8	D6, D7, F6, F4, H7, G7, F5, F3, E6	0.0291045
**May (D4)**		
0	A2	0.0255216
1	A2, B2	0.0252681
2	A2, B2, B3	0.0215447
3	A2, B2, B3, F1	0.0199938
4	A2, B2, B3, F1, D3	**0.0179418**
5	A2, B2, B3, F1, D3, H1	0.0181023
6	H7, D5, F7, A7, B7, B4, C7	0.0194512
**June (D5)**		
0	D4	0.0129441
1	D4, D3	**0.0105844**
2	D4, D3, C6	0.0109450
3	D4, D3, C6, C2	0.0110102
**July (D4)**		
0	D5	0.0152904
1	D5, F1	**0.0143873**
2	D5, F1, D6	0.0148891
3	D5, F1, D6, D3	0.0152913
**October (B7)**		
0	B1	0.0343254
1	B1, D3	0.0302872
2	B1, D3, B2	**0.0298292**
3	B1, D3, B2, F1	0.0312717
4	B1, D3, B2, F1, D2	0.0324157

***NB:** Sensor location in parenthesis implies the location with the least RMSE value - most important (predictor). The bolded values represent optimal sensor locations.*

A similar trend with the temperature data was seen in the Pareto fronts for the optimal number of sensors to accurately measure the air-vapor mixture in the greenhouse using the relative humidity data. A flat Pareto front was seen in the reduction of RMSE in February (Figure 8A) from 2 to 8 sensors. All other months (Figures 8B–G) showed that the Pareto front improved decision-making, as there were sensors that though reduced the RMSE, did not significantly cause the front to change.

The temperature and relative humidity data had the same predictor, implying the location with the least RMSE value (Tables 2, 3). This indicated that the rankings were not different (shows the sensors with the least interference for the month). However, the optimal numbers of sensors using the temperature and relative humidity data varied across the months.

### Transformed Data: Psychometric Variables

The optimal sensor locations for February, March, April, May, June, July, and October are given in Table 4 for the four psychrometric properties considered in this study. The monthly data was split into daily and weekly data to get a clearer view of optimal sensors placement for each month and investigate the effect of the sharp changes in weather conditions. Analyses of the sensor numbers results show that the transformed psychrometric variables had fewer optimal locations than the untransformed (temperature and relative humidity) dataset, with a difference of up to about 70% in May.

**TABLE 4:** Seasonal variation in optimal sensor placement.

February (Winter)					March (Spring)					April (Spring)					May (Spring)					June (Summer)					July (Summer)					October (Autumn)				
U	Td	w	h	v	U	Td	w	h	v	U	Td	w	h	v	U	Td	w	h	v	U	Td	w	h	v	U	Td	w	h	v	U	Td	w	h	v
F7	F7	F7	F7	F7	G7	G7	G7	G7	G7	F7	F7	F7	F7	F7	D4	D4	D4	D4	D4	D5	D5	D5	D5	D5	D4	D4	D4	D4	D4	B7	B7	B7	B7	B7
C2	C2	C2	C2	C2	H7	H7	H7	H7	H7	D6	D6	D6	D6	D6	A2	A2	A2	A2	A2	D4	D4	D4	D4	D4	D5	D5	D5	D5	D5	B1	B1	B1	B1	B1
H7	H7	H7	H7	H7	D5	D5	D5	D5	D5	D7	D7	D7	D7	D7	B2	B2	B2	B2	B2	D3	D3	D3	D3	D3	F1	F1	F1	F1	F1	D3	D3	D3	D3	D3
		B7	B7	B7	F7	F7	F7	F7		F6	F6	F6	F6	F6	B3	B3	B3	B3	B3	C6	C6	C6	C6	C6	D6	D6	D6	D6	D6					
			A1	A1	A7	A7	A7	A7		F4		F4	F4	F4	F1	F1	F1	F1	F1	C2					D3	D3	D3	D3	D3					
			D6	D6	B7	B7	B7	B7		H7		H7	H7	H7	D3		D3	D3		C4					C4	C4	C4	C4	C4					
					B4	B4	B4			G7		G7	G7	G7	H1		H1	H1							D2	D2	D2	D2	D2					
					C7	C7	C7			F5		F5			A4		A4	A4							C6	C6	C6	C6	C6					
					E7	E7	E7			F3		F3			C4		C4	C4							F7	F7	F7	F7	F7					
						D7	D7			E6		E6			A3		A3	A3								E1	E1	E1	E1					
						D4	D4			E7		E7			D2											H7								
										H6		H6			C2											F4								
										D2		D2			E1											F5								
												D5			C1																			
															A6																			
															C3																			
															B1																			
(3)	(3)	(6)	(6)	(4)	(9)	(11)	(11)	(6)	(3)	(13)	(4)	(14)	(7)	(7)	(17)	(5)	(10)	(10)	(5)	(6)	(4)	(4)	(4)	(4)	(9)	(13)	(10)	(10)	(10)	(3)	(3)	(3)	(3)	(3)

*Keys: U – untransformed (raw temperature and humidity) data. Td – dew point temperature. w – humidity ratio. h – enthalpy. v - specific volume.*

**FIGURE 9:**
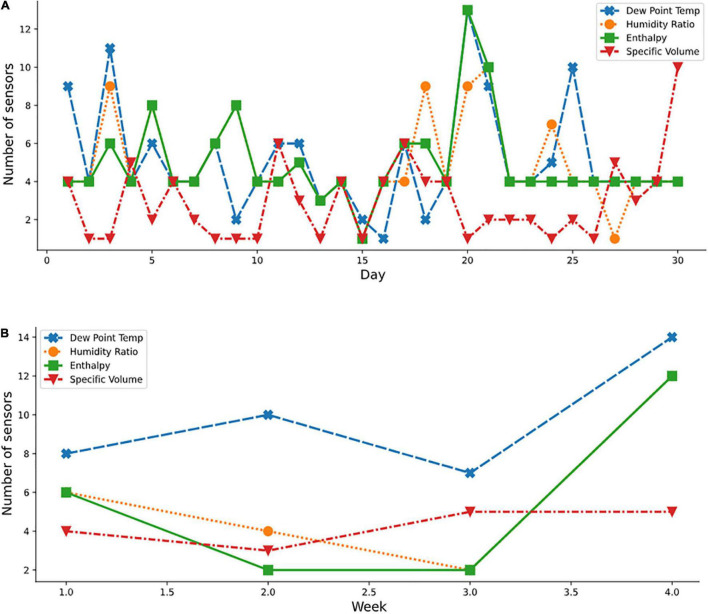
Optimal sensor selection for spring month using the psychometric dataset; **(A)** April daily; and **(B)** weekly.

Figures 9A,B show the daily and weekly distributions of the variables by sensors for a spring month (April), respectively. Using the transformed psychometric properties (dew point temperature, humid ratio, enthalpy, and specific volume), inconsistencies in selections of the ideal number of sensors required for some days in April were observed. For instance, on day 1 (April), nine optimal sensors were required when the dew point temperature property was considered, while four optimal sensors were required for other properties. The usage of the derived psychometric properties resulted in the selection of a reduced number of optimal sensors indicating a more adaptive nature of the algorithm to these derived variables compared to the raw temperature and relative humidity variables. The derived psychometric properties also showed better understanding of the air-vapor mixture since two combined properties were considered instead of the untransformed dataset using a single property. This improved efficiency would benefit the grower by reducing acquisition and operating costs as well as decreasing amounts of dat ato be handled. Furthermore, a cross-cutting beneficial effect would result from energy savings from proper monitoring and increased productivity.

Over the study period, specific volume (v) required the least number of sensors for measurement. However, it showed the most inconsistent result (having values not in a close range with the result from other derived properties), likely due to the very low magnitude of the values producing slightly more stochastic predictions.

**FIGURE 10:**
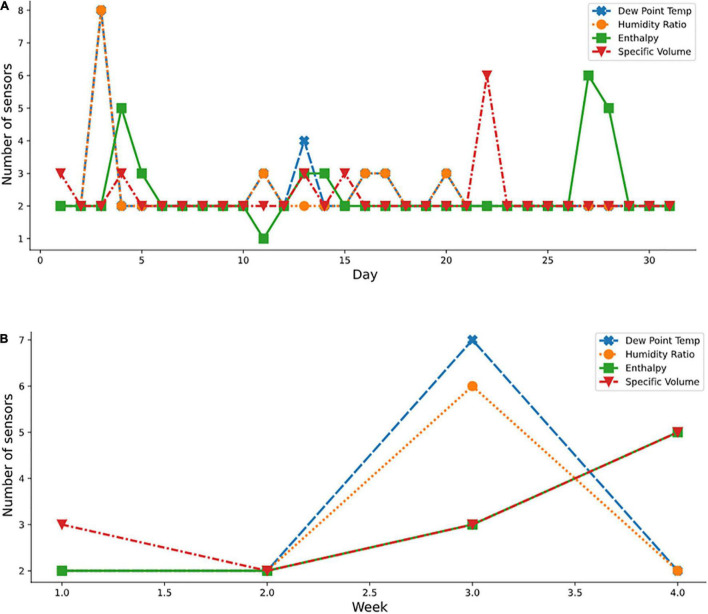
Optimal sensor selection for summer month using the psychometric dataset; **(A)** July daily; and **(B)** weekly.

**FIGURE 11:**
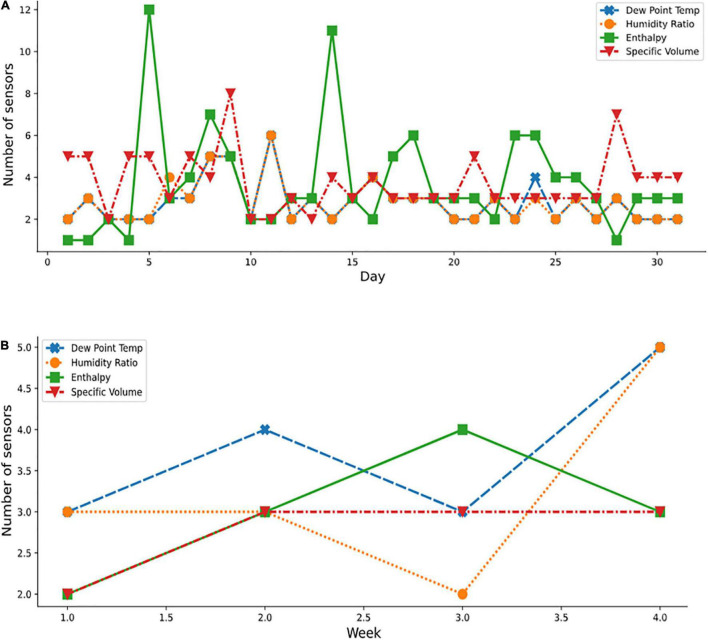
Optimal sensor selection for Autumn month using the psychometric dataset; **(A)** October daily; and **(B)** weekly.

Additionally, it was noted that the order of sensor selection did not change over the study period. For example, as reported for April in Table 4, in the April column, the ranking of the 13 sensor locations according to decreasing order of importance was F7, D6, D7, F6, F4, H7, G7, F5, F3, E6, E7, H6, and D2. If four sensors were required for measuring the enthalpy variable, then the first four sensor locations (F7, D6, D7, and F6) were to be considered. If one sensor only was selected, then F7 was the optimal sensor. Figures 10, 11 show the periodic variation of optimal sensor selection for summer (July) and autumn (October), respectively.

### Periodic Variation in the Optimal Sensor Selection

Several plots (Figures 9–11) show the variation in the optimal sensor selection for the greenhouse over time (that is, daily and weekly). External disturbances such as temperature, wind, and humidity influenced the data. Modeling the phenomenon of natural ventilation proved to be complex, especially because it was significantly affected by the external climate, and its design more complicated than fan ventilation. A fan ventilation system was adopted for verification (Figure 3C). Yet, significant variations were still observed based on the analysis of the coefficient of variation of the indoor climate data (Table 1). Also thought to be influencing the microclimate within the protected cultivation systems were factors such as the heating system and the respiration of the plants which could have led to variations in the relative humidity. This necessitated a systematic approach for determining the optimal number and locations of the sensors. For example, on days 15 and 20 of April (Figure 9A), temperature measurements and standard deviations of 0.3258°C and 0.2130°C, respectively were reported. Statistically, in terms of measuring dispersion, the magnitude of the standard deviation varied across daily, weekly, and monthly periods. These temporal variations in the results indicated that optimal sensor placement was affected by periodic variations of different levels of magnitude. However, the sensors selected at the same level across the measured conditions and transformed psychrometric properties were the same, pointing to the robustness of our method to accurately measure the total air-vapor mixture in the protected cultivation system.

Additionally, the indoor heating system could contribute to the differences in the optimal locations selected to measure environmental conditions as some piping systems heat sections of the greenhouse nonuniformly.

## Conclusion

A supervised machine learning model was developed to identify the optimal number and locations of sensors to monitor climatic conditions in a protected cultivation system using a multi-objective approach. The Gradient Boosting Algorithm was fitted to the measured conditions and derived psychrometric variables. The derived psychrometric properties resulted in fewer optimal sensors than the raw temperature and relative humidity data. This study found that the optimal locations of sensors were both at the sides and center of the protected cultivation system depending on the time of year. Variability analyses indicated that no location was consistently optimal. The changes in the optimal sensor location with seasons were this study’s limitation. A future study would aim to develop a dynamic approach to selecting optimal sensors’ locations. This could include using the ensemble technique by creating multiple models and considering a mobile environmental measurement system. Finally, the solutions in the Pareto front improved decision-making as some points had close relationships. This would have cross-cutting effects on energy management and plant productivity.

## Data Availability Statement

The raw data supporting the conclusions of this article will be made available by the authors, without undue reservation.

## Author Contributions

DU: conceptualization, methodology, investigation, formal analysis, data curation, supervision, visualization, and writing – original draft. OI: methodology, investigation, software, data curation, visualization, and writing – original draft. RM: methodology, investigation, software, data curation, visualization, and writing – review and editing. SA-H: methodology, validation, data curation, visualization, supervision, and writing – review and editing. YH: validation, resources, writing – review and editing, and supervision. MA: methodology, investigation, and supervision. TP: methodology, investigation, software, data curation, visualization, validation, resources, writing – review and editing, supervision, and funding acquisition. All authors contributed to the article and approved the submitted version.

## Conflict of Interest

The authors declare that the research was conducted in the absence of any commercial or financial relationships that could be construed as a potential conflict of interest.

## Publisher’s Note

All claims expressed in this article are solely those of the authors and do not necessarily represent those of their affiliated organizations, or those of the publisher, the editors and the reviewers. Any product that may be evaluated in this article, or claim that may be made by its manufacturer, is not guaranteed or endorsed by the publisher.

## References

[B1] Arnesano M. Revel G. Seri F. (2016). A tool for the optimal sensor placement to optimize temperature monitoring in large sports spaces. *Autom. Constr.* 68 223–234. 10.1016/j.autcon.2016.05.012

[B2] Ayalew D. Tesfaye K. Mamo G. Yitaferu B. Bayu W. (2012). Variability of rainfall and its current trend in Amhara region, Ethiopia. *Afr. J. Agric. Res.* 7 1475–1486. 10.5897/AJAR11.698

[B3] Aydin B. E. Hagedooren H. Rutten M. M. Delsman J. Oude Essink G. H. van de Giesen N. (2019). A greedy algorithm for optimal sensor placement to estimate salinity in polder networks. *Water* 11:1101. 10.3390/w11051101

[B4] Bhujel A. Basak J. K. Khan F. Arulmozhi E. Jaihuni M. Sihalath T. (2020). Sensor systems for greenhouse microclimate monitoring and control: a review. *J. Biosyst. Eng.* 45 341–361. 10.1007/s42853-020-00075-6

[B5] Boerema S. T. Van Velsen L. Schaake L. Tönis T. M. Hermens H. J. (2014). Optimal sensor placement for measuring physical activity with a 3D accelerometer. *Sensors* 14 3188–3206. 10.3390/s140203188 24553085 PMC3958275

[B6] Chang D.-E. Ha K.-R. Jun H.-D. Kang K.-H. (2012). Determination of optimal pressure monitoring locations of water distribution systems using entropy theory and genetic algorithm. *J. Korean Soc. Water Wastewater* 26 1–12. 10.11001/jksww.2012.26.1.001

[B7] Chang M. Pakzad S. N. (2014). Optimal sensor placement for modal identification of bridge systems considering number of sensing nodes. *J. Bridge Eng.* 19:04014019. 10.1061/(ASCE)BE.1943-5592.0000594 29515898

[B8] Cossu M. Murgia L. Ledda L. Deligios P. A. Sirigu A. Chessa F. (2014). Solar radiation distribution inside a greenhouse with south-oriented photovoltaic roofs and effects on crop productivity. *Appl. Energy* 133 89–100. 10.1016/j.apenergy.2014.07.070

[B9] Czubinski F. F. Mantelli M. B. Passos J. C. (2013). Condensation on downward-facing surfaces subjected to upstream flow of air–vapor mixture. *Exp. Therm. Fluid Sci.* 47 90–97. 10.1016/j.expthermflusci.2013.01.004

[B10] DeFacio P. Pickerel L. Rhyne S. M. (2002). *Greenhouse Operation and Management: Instructional Materials Laboratory.* Columbia, MO: University of Missouri, 10.

[B11] Du W. Xing Z. Li M. He B. Chua L. H. C. Miao H. (2014). “Optimal sensor placement and measurement of wind for water quality studies in urban reservoirs. IPSN-14,” in *Proceedings of the 13th International Symposium on Information Processing in Sensor Networks*, (Piscataway, NJ: IEEE). 10.1109/IPSN.2014.6846750

[B12] Faris D. M. Mahmood M. B. (2014). Data acquisition of greenhouse using Arduino. *J. Babylon Univ.* 22 1908–1906.

[B13] Feng L. Li H. Zhi Y. (2013). “Greenhouse CFD simulation for searching the sensors optimal placements,” in *Proceedings of the 2013 Second International Conference on Agro-Geoinformatics (Agro-Geoinformatics)*, (Piscataway, NJ: IEEE). 10.1109/Argo-Geoinformatics.2013.6621972

[B14] Fontanini A. D. Vaidya U. Ganapathysubramanian B. (2016). A methodology for optimal placement of sensors in enclosed environments: a dynamical systems approach. *Build. Environ.* 100 145–161. 10.1016/j.buildenv.2016.02.003 32287963 PMC7126557

[B15] Furlanello C. Merler S. Jurman G. (2006). Combining feature selection and DTW for time-varying functional genomics. *IEEE Trans. Signal Process.* 54 2436–2443. 10.1109/TSP.2006.873715

[B16] Gadekallu T. R. Rajput D. S. Reddy M. Lakshmanna K. Bhattacharya S. Singh S. (2021). A novel PCA–whale optimization-based deep neural network model for classification of tomato plant diseases using GPU. *J. Real Time Image Process.* 18 1383–1396. 10.1007/s11554-020-00987-8

[B17] Graamans L. Baeza E. Van Den Dobbelsteen A. Tsafaras I. Stanghellini C. (2018). Plant factories versus greenhouses: comparison of resource use efficiency. *Agric. Syst.* 160 31–43. 10.1016/j.agsy.2017.11.003

[B18] Guzmán C. H. Carrera J. L. Durán H. A. Berumen J. Ortiz A. A. Guirette O. A. (2019). Implementation of virtual sensors for monitoring temperature in greenhouses using CFD and control. *Sensors* 19:60. 10.3390/s19010060 30586913 PMC6339024

[B19] Handbook A. (2001). *Fundamentals, 2001.* Atlanta: ASHRAE.

[B20] Hemming S. Zwart F. D. Elings A. Petropoulou A. Righini I. (2020). Cherry tomato production in intelligent greenhouses—sensors and AI for control of climate, irrigation, crop yield, and quality. *Sensors* 20:6430. 10.3390/s20226430 33187119 PMC7698269

[B21] Hu J. Patel M. (2014). “Optimized selection and placement of sensors using building information models (BIM),” in *Proceedings of the IES Annual Conference*, Pittsburgh, PA.

[B22] Huang G. Zhou P. Zhang L. (2014). “Optimal location of wireless temperature sensor nodes in large-scale rooms,” in *Proceedings of the 13th International Conference on Indoor Air Quality and Climate, Indoor Air*, Hong Kong.

[B23] Jones M. B. (1985). “Chapter 3 – plant microclimate,” in *Techniques in Bioproductivity and Photosynthesis*, 2nd Edn, eds Coombs J. Hall D. O. Long S. P. Scurlock J. M. O. (Oxford: Pergamon Press), 26–40. 10.1016/B978-0-08-031999-5.50013-3

[B24] Kader A. A. Saltveit M. E. (2002). *Respiration And Gas Exchange: Postharvest Physiology And Pathology Of Vegetables.* Boca Raton, FL: CRC Press, 31–56. 10.1201/9780203910092.ch2

[B25] Kassie B. T. (2014). *Climate variability And Change In Ethiopia: Exploring Impacts And Adaptation Options For Cereal Production.* Wageningen: Wageningen University and Research.

[B26] Lee S.-Y. Lee I.-B. Yeo U.-H. Kim R.-W. Kim J.-G. (2019). Optimal sensor placement for monitoring and controlling greenhouse internal environments. *Biosyst. Eng.* 188 190–206. 10.1016/j.biosystemseng.2019.10.005

[B27] Li C. (2016). *A Gentle Introduction To Gradient Boosting.* online at: https://www.ccs.neu.edu/home/vip/teach/MLcourse/4_boosting/slides/gradient_boosting (accessed December 13, 2020).

[B28] Li S. Cheng X. Chen Y. Zhang H. (2013). The optimal placement of sensors in square target regions with varying boundary length. *Proc. Eng.* 62 899–906. 10.1016/j.proeng.2013.08.141

[B29] Li X.-H. Cheng X. Yan K. Gong P. (2010). A monitoring system for vegetable greenhouses based on a wireless sensor network. *Sensors* 10 8963–8980. 10.3390/s101008963 22163391 PMC3230972

[B30] Löhner R. Camelli F. (2005). Optimal placement of sensors for contaminant detection based on detailed 3D CFD simulations. *Eng. Comput.* 22 260–273. 10.1108/02644400510588076

[B31] Nelson P. V. (1991). *Greenhouse Operation And Management.* Hoboken, NJ: Prentice Hall.

[B32] Pamungkas A. P. Hatou K. Morimoto T. (2014). Evapotranspiration model analysis of crop water use in plant factory system. *Environ. Control Biol.* 52 183–188. 10.2525/ecb.52.183 21291192

[B33] Park D.-H. Park J.-W. (2011). Wireless sensor network-based greenhouse environment monitoring and automatic control system for dew condensation prevention. *Sensors* 11 3640–3651. 10.3390/s110403640 22163813 PMC3231320

[B34] Park S.-H. Park T. Park H. D. Jung D.-H. Kim J. Y. (2019). Development of wireless sensor node and controller complying with communication Interface standard for smart farming. *J. Biosyst. Eng.* 44 41–45. 10.1007/s42853-019-00001-5

[B35] Ponce P. Molina A. Cepeda P. Lugo E. MacCleery B. (2014). *Greenhouse Design And Control.* Boca Raton, FL: CRC Press. 10.1201/b17391

[B36] Prieto I. Armas C. Pugnaire F. I. (2012). Water release through plant roots: new insights into its consequences at the plant and ecosystem level. *New Phytol.* 193 830–841. 10.1111/j.1469-8137.2011.04039.x 22250761

[B37] Rao A. R. M. Lakshmi K. Krishnakumar S. (2014). A generalized optimal sensor placement technique for structural health monitoring and system identification. *Proc. Eng.* 86 529–538. 10.1016/j.proeng.2014.11.077

[B38] Seabrook T. (2016). *Optimal Placement Strategies of Minimum Effective Sensors for Application in Smart Buildings.* online at: https://www.semanticscholar.org/paper/Optimal-Placement-Strategies-of-Minimum-Effective-Seabrook/ [Accessed December 10, 2018].

[B39] Stanghellini C. (2013). “Horticultural production in greenhouses: efficient use of water,” in *Proceedings of the International Symposium on Growing Media and Soilless Cultivation*, Leuven, 1034. 10.17660/ActaHortic.2014.1034.1

[B40] Syed A. M. Hachem C. (2019a). Review of design trends in lighting, environmental controls, carbon dioxide supplementation, passive design, and renewable energy systems for agricultural greenhouses. *J. Biosyst. Eng.* 44 28–36. 10.1007/s42853-019-00006-0

[B41] Syed A. M. Hachem C. (2019b). Review of construction; geometry; heating, ventilation, and air-conditioning; and indoor climate requirements of agricultural greenhouses. *J. Biosyst. Eng.* 44 18–27. 10.1007/s42853-019-00005-1

[B42] Tong K. Bakhary N. Kueh A. Yassin A. (2014). Optimal sensor placement for mode shapes using improved simulated annealing. *Smart Struct. Syst.* 13 389–406. 10.12989/sss.2014.13.3.389

[B43] Uyeh D. D. Pamulapati T. Mallipeddi R. Park T. Asem-Hiablie S. Woo S. (2019). Precision animal feed formulation: an evolutionary multi-objective approach. *Anim. Feed Sci. Technol.* 256:114211. 10.1016/j.anifeedsci.2019.114211

[B44] Uyeh D. D. Pamulapati T. Mallipeddi R. Park T. Woo S. Lee S. (2021). An evolutionary approach to robot scheduling in protected cultivation systems for uninterrupted and maximization of working time. *Comput. Electron. Agric.* 187:106231. 10.1016/j.compag.2021.106231

[B45] Vox G. Teitel M. Pardossi A. Minuto A. Tinivella F. Schettini E. (2010). *Sustainable Greenhouse Systems: Sustainable Agriculture: Technology, Planning And Management.* New York, NY: Nova Science Publishers Inc, 1–79.

[B46] Wang X. Ma J. Wang S. (2009). Parallel energy-efficient coverage optimization with maximum entropy clustering in wireless sensor networks. *J. Parallel Distrib. Comput.* 69 838–847. 10.1016/j.jpdc.2009.04.012

[B47] Wang X.-H. Xu L.-H. Wei R.-H. (2014). “A new fusion structure model on greenhouse environment data and a new fusion algorithm of sunlight,” in *Proceedings of the 2014 International Conference on Wireless Communication and Sensor Network*, (Piscataway, NJ: IEEE). 10.1109/WCSN.2014.91

[B48] Worden K. Burrows A. (2001). Optimal sensor placement for fault detection. *Eng. Struct.* 23 885–901. 10.1016/S0141-0296(00)00118-8

